# Spondylodiscitis: experience of surgical management of complicated cases after failed antibiotic treatment

**DOI:** 10.1051/sicotj/2020002

**Published:** 2020-02-14

**Authors:** Abdullah Mohammed Kamal, Mohammad M. El-Sharkawi, Moataz El-Sabrout, Mohammad Gamal Hassan

**Affiliations:** 1 Department of Orthopaedic and Trauma Surgery, Faculty of Medicine, Assiut University Assiut 71511 Egypt

**Keywords:** spondylodiscitis, spinal infection, spinal fusion, pseudarthrosis, multi-disciplinary approach

## Abstract

*Introduction*: The term Spondylodiscitis (SD) involves infection of the vertebra (Spondylitis), infection of the intervertebral disc (Discitis), or both (Spondylodiscitis). SD represents a diagnostic and therapeutic challenge to any spine surgeon. Any delay in its diagnosis or management may cause serious long-term morbidity or even lead to mortality. In this study, we report the experience of our Institution in the management of severe and complicated cases of SD. *Methods*: Over a period of 1 year, 39 patients with the diagnosis of SD were surgically treated in Assiut University Hospital, Assiut, Egypt. The management processes were tailored according to the clinical condition, radiological and lab studies of each case; and patients were then prospectively followed-up until they were cured (for a minimum of 6 months). The outcomes were analyzed, to be able to give recommendations while aiming to improve the overall outcome of such dangerous health issue. *Results*: In this series, patients were managed surgically by drainage and debridement of the infection site with/without instrumented fusion. Results included: satisfactory fusion was achieved in 97.3% of patients (confidence interval [CI] = 0.6856–1.3421). Neurological Improvement Rate (NIR) was 71.5% (Statistically significant improvement *P*-value = 0.014) and reoperation rate was 5% (CI = 0.00621–0.18525). Mortality rate was 7.7% (CI = 0.016–0.209). Several aspects were analyzed in each case. *Conclusion*: Surgical management of severe and complicated cases of SD allows for effective debridement and rapid cure of inflammation, earlier patient mobilization and significantly shorter duration of antibiotic usage.

## Introduction

Spinal infection accounts for 2–7% of all cases of musculoskeletal infections [1]. Pathogens can reach the spine either by: hematogenous spread, direct external inoculation, or spread from contiguous tissues harboring these pathogens. The hematogenous route is the predominant one, allowing seeding of infection from distant sites into the vertebral column [2].

There are a variety of organisms that are involved in spine infections, including bacteria, fungi, and parasites. *Staphylococcus aureus* represents the predominant pathogen (20–84%); accounting for about half of non-tuberculous cases. *Mycobacterium tuberculosis* still represents a major cause of spinal infections, being responsible for about 9–46% of spinal infections [2].

In its early clinical stages, *mild* [3] spondylodiscitis (SD) (early presenting cases, not complicated with neurological deficit, and/or advanced bony destruction, and the patient had not have failed medical treatment) responds favorably to conservative therapy, consisting mainly of antimicrobial chemotherapy and immobilization [3]. However, as the pathological process becomes more progressive, through spread of the infection, complications occur as appearance and worsening of both skeletal deformity and neurological deficits, as well as possibility of body sepsis (severe SD [3]), it becomes highly potential that conservative management would fail, and therefore, surgery – still combined with adjunct suitable antibiotic treatment – becomes inevitably indicated [4], in order to debride the primary infected tissue, obtain specimens for microbiological testing and/or histopathological examination, decompression of spinal canal, as well as bony fusion to achieve bony stability [5].

Despite the above mentioned rules, the course of action while managing SD stays an area of debate among major Spine Centers around the world, because of the very inhomogeneous group of its patients [6], the similar rates of morbidity and mortality following both conservative and surgical treatment [7] and the suggestion that surgical treatment in uncomplicated spondylodiscitis could result in faster recovery, faster mobilization, and a better short-term quality of life when compared to conservative treatment [8].

*The aims of this study* were to report our institution experience in the management of patients with severe and complicated SD, initially poorly managed elsewhere with the use of empirical antibiotics before their referral to our institute, to provide analysis of the patients in this series, comparing several aspects in the multiple management plans we provided; and interpreting their postoperative recovery course as well as outcome results, and to correlate our findings with those of other authors, to identify points of strengths and weaknesses of current management strategies, while give final recommendations accordingly, to improve the quality of care given to patients with challenging SD.

## Patients and methods

This prospective case series was performed in Assiut University Hospital, Assiut, Egypt, on patients with SD admitted to the Orthopedic and Trauma Surgery Department, during a period of one year; from January to December of 2016. Assiut University Hospital is a large Academic Tertiary Referral Center that serves a substantial portion (about a third) of the Egyptian population. Our criteria for hospital admission of patients with SD were one or more of the following: (1) presence of medical comorbidity(ies), presence of: (2) neurological impairment, (3) fever, and/or (4) sepsis on presentation, (5) elderly patients, (6) imaging (plain X-rays or computerized tomography [CT]) evidence of significant bony destruction and instability and/or presence of epidural abscess on magnetic resonance imaging (MRI) with or without neurological elements compromise, and (7) patients with persistent SD despite lengthy conservative management. All the patients who underwent surgery during that period were included in this study (39 patients). This study was approved by our Institution Review Board (IRB number: 17100807) and all patients signed an informed consent.

Each SD case is discussed in detail by all Faculty Members of our Spine Unit (12 Spine Surgery Consultants) during our daily case-discussion meetings; and the management then is decided by consensus and general collective agreement. In addition, in patients who have associated other health issue(s), which could potentially impact the management and/or outcome of their SD, our general practice involves addressing these co-morbid conditions immediately following hospital admission, and prior to surgery. This is accomplished through *multi-disciplinary team approach* via consulting providers from other specialties including Infectious Diseases specialists, Nephrologists, Gastroentrologists, Cardiologists, Internists, or Anesthesiologists, in order to control these issues and/or ensure the patient’s fitness for the surgery. This team-approach is applied in managing our patients in throughout the preoperative, perioperative, and postoperative periods and clinic follow-up visits, while intending to improve the overall outcomes of managing patients with SD. The surgical procedures were afterward carried out by experienced spine surgeons, and it consisted of thorough debridement of infected tissues, obtaining samples for histopathological examination and/or culture and sensitivity testing, with or without decompression of neural structures, or bony fusion.

Being a tertiary healthcare center, all patients were referred to us already on antibiotic treatment. Patients were continued on empirical intravenous antibiotics postoperatively during their hospital stay and were switched to oral antibiotics on their discharge until the results of histopathology and/or culture and sensitivity became available. By that time, they were finally put on suitable oral antibiotics, the type and duration of which were determined by the results of the aforementioned investigations.

**Figure 1:**
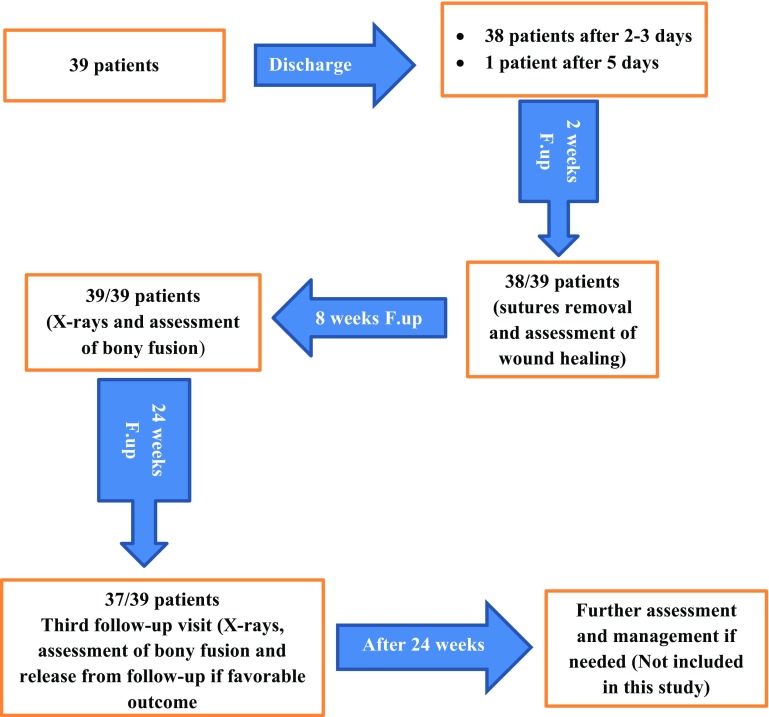
Flow diagram for follow-up.

The majority of patients are discharged within the first 72 h postoperatively; unless complications occur, or the general medical condition is not stable enough to allow safe discharge. Neurological status is checked and appropriately recorded frequently for all study patients, including: on admission, immediately postoperatively, in every single day while the patient is still admitted in the hospital, as well as, on discharge and in every outpatient follow-up visit afterward. By the time of release from the hospital, all patients are scheduled for a minimum of three postoperative standard regular outpatient clinic follow-up visits, at 2 weeks, at 8 weeks, and at 6 months postoperatively (Flow diagram, Figure 1). Further outpatient follow-up visits, whether within and/or after these first 6 months postoperative period, are decided and carried out according to the progress of each individual case throughout the recovery stage.

*Clinical outcomes* were evaluated using Visual Analogue Scale (VAS) [9] for back pain, and American Spinal Injury Association (ASIA) Impairment score [10] for neurological evaluation. Radiological assessment included fusion state, position of cage/graft, and position of posterior instrumentation. Statistical analysis of the data was performed using SPSS 17.0 software (SPSS Inc., Chicago, IL). For continuous values (such as age), the mean was calculated to derive the central tendency of the data. Grouped values (such as preoperative and postoperative neurological status) were evaluated using a Pearson chi-square test; values of less than 0.05 were considered significant.

## Results

From January to December 2016, 39 consecutive patients who underwent surgery for the treatment of SD were included in this study and were prospectively followed-up for a minimum of 6 months – until they were cured.

The study cohort included 24 males (61.5%) and 15 females (38.5%), with a mean age of 56 years, and 26 (66.6%) of them had associated risk factors for SD, in the form of either medical comorbidities (25 patients, 64%) or previous spine surgery (1 patient, 2.6%) (Table 1). *Multidisciplinary team approach* was applied in the management of multi-morbid patients.

**Table 1:** Risk factors*.

Risk factor	Patients (%)
Bronchial asthma	1 (2.5)
Chronic kidney disease (CKD)	12 (30.7)
Diabetes mellitus	5 (12.8)
Chronic hepatic impairment	11 (28.2)
Hypertension	5 (12.8)
Ischemic heart disease	1 (2.5)
Previous spine surgery	1 (2.5)
None	14 (35.9)

* Patients may have more than one risk factor.

All patients in this study had presented with neck/back pain; while, fever was only found in 10 patients (25.6%). In addition, a total of 21 patients (21/39, 53.8%) had various degrees of neurological impairment on presentation.

The spine segments affected with SD, as identified by X-rays and MRI, were as follows: cervical (2/39, 5%), thoracic (16/39, 41%), thoraco-lumbar junction (6/39, 15.4%), lumbar and lumbo-sacral (15/39, 38.6%). Imaging results included: single-level infection (infection of a single disc and the two adjacent vertebrae) in 35 patients (89.7%), two levels in 4 patients (10.3%). We did not have any case of isolated spondylitis or isolated discitis in this period.

In this study, the surgical intervention ranged from just evacuation of epidural abscess(es) associated with debridement of necrotic tissues, all through to combined anterior debridement and decompression and posterior instrumentation.

**Figure 2:**
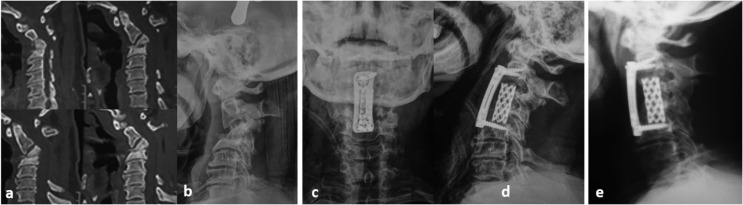
SD C3–4 in a 62-year-old male. (a) Preoperative CT scan showing C3–4 SD with significant local kyphosis. (b) Preoperative lateral X-rays after traction for 2 weeks. (c and d) Postoperative X-rays anteroposterior and lateral views showing corpectomy of C3 and C2–4 fusion using titanium cage and cervical locked H-shaped plate. (e) Six months follow-up lateral X-rays showing solid fusion.

**Figure 3:**
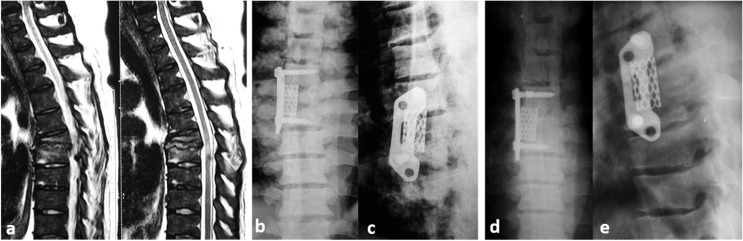
SD D6–7 in a 47-year-old male. (a) Preoperative sagittal MRI veiws. (b and c) Immediate postoperative X-rays showing cropectomy of D6 and fusion D5–7 using titanium cage and plate via anterior approach. (d and e) Follow-up X-rays after 6 months.

**Figure 4:**
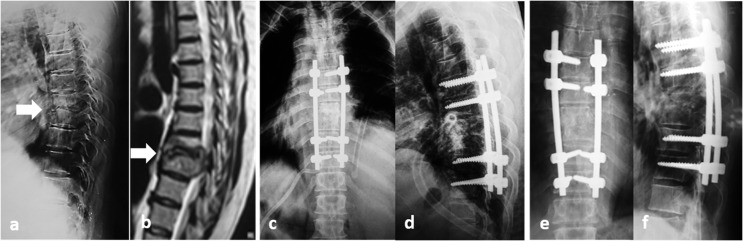
SD D8–9 in a 58-year-old female. (a and b) Preoperative lateral X-rays and MRI sagittal veiws. (c and d) Immediate postoperative X-rays showing partial corpectomy D8–9 with interbody fusion using bone garft and long segment pedicular screw fixation from D6–11 (Posterior approach). (e and f) Follow-up X-rays after 6 months.

The surgical approach used is usually dictated by the anatomic location of the infection (cervical/thoracic/lumbar), the degree of bony destruction, the presence or absence of a deformity, and the expertise and preference of the surgeon. Two patients with cervical SD were approached anteriorly from the right side; and both underwent corpectomy of the affected level, along with fusion by cage and locked plate (Figure 2). The other 37/39 patients who had thoracic, thoracolumbar as well as lumbar SD fell into one of three groups according to the surgical approach utilized: The *Anterior*-*only* transthoracic or retroperitoneal approach (5 patients), using titanium cages and plates for stabilization (Figure 3); The *Posterior-only* approach (31 patients) where drainage, decompression, and debridement were done in all patients and additional stabilization and fusion in 30 patients through a transforaminal interbody fusion (21/30 patients) or a posterolateral corpectomy (9/30 patients) and either tricortical iliac crest graft mixed with cancellous bone chips (12/30 patients) (Figure 4) or a suitably sized cage filled with cancellous bone graft (18/30 patients); both techniques had been thoroughly described in a previous publication [11]; The *Combined Anterior* decompression and debridement and *Posterior* instrumentation (Table 2) which included 1 patient.

**Table 2:** Approaches utilized in thoracic and/or Lumbar SD.

Approach	Patients
Posterior	
Debridement and fusion	30
Debridement only	1
Anterior	5
Combined (anterior and posterior)	1
Total	37

Biopsy was obtained intraoperatively for histopathological examination in all 39 patients, and the results of which were: 32 patients (32/39) had chronic non-specific inflammation (82%), while 7 patients (7/39) had caseating granulomatous inflammation (18%). Culture and sensitivity examination was also done for all cases, but was positive only in four cases.

All patients were maintained on broad spectrum intravenous antibiotic, *Ceftriaxone* postoperatively during their hospital admission. On discharge, they were shifted to oral antibiotics, *Linezolid* and *Ciprofloxacin* until the results of histopathology and/or culture and sensitivity became available. Seven patients had chronic caseating granulomatous inflammation on their histopathological examination results – the classic picture of tuberculous infection [12] was treated with anti-tuberculous combination therapy (*Rifampicin*, *Izoniazide* and either *Ethambutol*, *Pyrazinamide,* or *Streptomycin*) for 6 months [13]. Only 4 out of 32 patients with histopathological examination results confirming chronic non-specific inflammation had positive culture and sensitivity results and these were shifted to oral organism-specific antibiotics (Table 3). Culture and sensitivity results could not reveal the offending organism in 28 patients, and based on their histopathological examination results of chronic non-specific inflammation, they were continued on oral combination of *Linezolid* and *Ciprofloxacin*. The total duration of postoperative antibiotic management was 8 weeks.

**Table 3:** Results of culture and sensitivity.

Isolated organism	Antibiotic
Klebsiella	Gentamycin
Gram-positive Bacteria (MRSA)	Imipenem
Actinobacter	Ofloxacin
Enterobacter & Enterococci	Augmentin

Concerning the outcome assessment of our study patients, overall management yielded satisfactory results, in the form of: (1) significant improvement of VAS score from an average of 8.2 (±0.76) preoperatively, to an average of 1.73 (±1.22) on last follow-up (*P*-value < 0.001), (2) *satisfactory surgical fusion* was achieved in 37 out of 38 patients (97.3%, Confidence Interval [CI] = 0.6856–1.3421), (3) *Neurological improvement* – by at least one point on ASIA grading system – was achieved in 15/21 patients (when comparing their grade in the last follow-up visit with that on original presentation); with Neurological Improvement Rate (NIR) of 71.5% (*P*-value = 0.014, *Table 4*) and (4) no recurrence of infection was recorded during our minimum six months follow-up period.

**Table 4:** Summary of neurology status on presentation and neurological improvement.

Neurology on presentation	Patients (39)	Improved (%)	Same as preoperative (%)	Worsened (%)
ASIA B	1	1 (100)	0 (0)	0
ASIA C	7	6 (86)	1 (14)	0
ASIA D	13	8 (61.5)	5 (38.5)	0
ASIA E	18	0 (0)	17 (94.4)	1 (5.6)*

* Patient A mentioned in details under the title of “Complications” in the Results section.

### Complications

(1) *Mortality*: Three patients died in the first 6 months postoperatively (7.7% mortality rate, CI = 0.016–0.209). These 3 patients included: patient A; this patient had L3-4 SD and underwent *Posterior* debridement and interbody fusion with transpedicular screws and bone graft. He suffered from multiple comorbidities including End-Stage Kidney Disease and was on dialysis. His discharge from the hospital was delayed for 5 days to allow postoperative stabilization of general condition upon which his VAS score had improved form 8 preoperatively to 3 postoperatively (on discharge) and his neurological status was not altered postoperatively (ASIA E). He presented after 8 weeks with deteriorated general condition, both VAS score (from 3 back up again to 9) and ASIA grade (changed from E to C). Upon that second presentation, immediate imaging confirmed failure of implant with progressive bony destruction. At that time, a decision was made for hospitalization, with the plan of stabilizing his general condition, and re-operation as soon as surgery is tolerable. The procedure was then performed using the same *Posterior* approach; through which debridement and longer segment fusion was done. It is to be noted that while this patient was recovering from anesthesia of this re-operative procedure, he demonstrated unstable vital signs and rapid deterioration of his general condition that required immediate transfer to the Intensive Care Unit (ICU). The course was afterward characterized by rapid worsening of his condition. All multi-disciplinary as well as ICU team efforts to rectify his general medical condition had failed; and the patient had unfortunately died in the second postoperative day. In addition, 2 other patients (patient B and C) who both shared common features including: both were elderly (76 and 74, respectively); and both had suffered from several medical comorbidities; where patient B had advanced degree of chronic hepatic function impairment; while patient C had diabetes mellitus, ischemic heart disease, and previous cerebrovascular stroke. They had thoracic SD and were approached *posteriorly*. Both patients showed satisfactory improvement in their standard outpatient postoperative follow-up visits. Death occurring at 3 months (patient B) and 6 months (Patient C) postoperatively was not directly related to their healing spondylodiscitis nor their surgery.

(2) *Wound healing problems* occurred in 1 patient (patient D) who originally underwent only drainage of an epidural abscess and debridement of infected/necrotic tissue through *Posterior* approach without bony fusion. On his first postoperative follow-up visit (2 weeks postoperatively), clinical examination suspected persistence of infection; however, plain X-rays showed no bony involvement. Therefore, reoperation was immediately decided, and very soon performed utilizing more thorough drainage and debridement techniques for all tissues believed by the surgeon to be necrotic or harbor infection. This patient was 44 years old and had no general risk factors; so, the possible cause of persistence of infection was highly suspicious to have been inefficient first operation technique. Of note, the overall reoperation rate was 5% (2/39) (CI = 0.00621–0.18525).

## Discussion

Conservative management with organism-specific antibiotics for prolonged period and spinal immobilization represents the first line treatment of SD, especially in early and mild cases. These cases are managed routinely in our institute on an outpatient basis as they do not meet our criteria for hospital admission; and therefore, were not included in this study. This study was carried out in the Orthopaedic Department of Assiut University Hospital, a very large Tertiary Teaching Healthcare Institution, in Assiut, Egypt, that drains millions of Upper Egyptian population, and focused only on complicated and resistant cases of spondylodiscitis. Most of the SD patients are referred to our institute while they are already in an advanced stage of their disease process, and likely with clear indications for surgery. In this study, this included advanced age of patients (mean age = 56 years) as well as the presence of several associated other major health issues in many of these patients (64%). These findings that we regularly encounter tended to limit the option of conservative management using antibiotics for extended periods of time, due to: (1) suboptimal function of body organs that decreases the potential of antimicrobial agent for accessing and eradicating an infected focus, (2) the compliance of the patients to the methods of external immobilization may be lacking and (3) possible increased risk of the side effects while administering longer term broad-spectrum antibiotics [14]. In comparison to conservative therapy, surgery allows for a safer and more rapid cure of the inflammation, and mobilization can be started relatively earlier after surgery [5].

The imaging modalities considered in this study were: (1) Plain X-ray, which represents the standard method and was done to all included patients in this study. It has a reported sensitivity of 82%, specificity of 57%, and accuracy of 73% [15]. It was done also for all patients on follow-up. Also, (2) MRI, the modality of choice for diagnosis of SD [15]. It has a reported sensitivity of 96%, specificity of 93%, and accuracy of 94% [16] and was also mandatory for all patients prior to establishment of diagnosis. On the other hand, (3) CT was done only in selected cases (Figure 2) to detect extent of bony destruction and aid in preoperative planning.

Regardless of the surgical approach used, the surgical goals include aggressive tissue debridement, sample harvesting for microbiology and histologic analyses, decompression of the spinal canal, drainage of paravertebral abscesses, restoration of spinal alignment, and stabilization by instrumentation and fusion [5]. The choice of surgical approach is not yet standardized, and is mostly based on local preferences resulting in physician-related variability [17, 18]. In our center, *posterior* approach is preferable by the majority of surgeons, so the number of patients in this study treated surgically utilizing this approach in thoracic and/or lumbar SD (32 patients) was higher than other approaches (*anterior only* 5 patients and *combined* approach one patient). Successful results using this approach were similar to previous studies [19, 20] regarding improvement in VAS score and achievement of bony fusion (30 of 31 patients, 96.7%).

Two patients with cervical SD underwent anterior surgery. The clear advantage of anterior instrumentation is better correction of the deformity [21]. The use of locked plate and titanium mesh cage (TMC) for reconstruction of bony defect following debridement and decompression provides a mechanically sound construct to achieve bony stability and maintain the correction [22].

In our study, there were 21 patients that had neurological impairment on presentation; 15 of them were improved by at least one grade on ASIA scale. In addition, NIR improvement of 71.5% was also in line with results stated by other authors [23, 24]. The improvement we encountered was found to be statistically significant (*P*-value 0.014).

Various previous studies found that culture and sensitivity results may be negative in up to 70% of cases of disc space infection (range from 30 to 70%) [25–27], which may be due to a variety of factors including that many of these patients get to Tertiary Referral Centers already on antibiotics, the fact that may confound the results of such a helpful test. Despite the fact that only 4 out of 39 patients in our study had positive culture and sensitivity test (10.2%), the results of histopathological examination were successfully used as an indirect guide for diagnosis and subsequent course of antimicrobial chemotherapy.

Histopathological examination of specimens obtained during surgery was done to all 39 surgically treated patients. It has been reported that this examination has high specificity (75–100%) and sensitivity (81–90%) in the diagnosis of SD [28–30]. It is also valuable in distinguishing between pyogenic and granulomatous SD and also in differential diagnosis between SD and other causes of vertebral destruction; mainly *malignancy* [1]. The fact that 7 (17.5%) patients had a final histopathological examination results of caseating granuloma (classically from TB infection) supports the suggestion that despite its global regression over the past few decades, TB is still a main cause of bone and joint infection in developing countries [31, 32].

Although CT-guided biopsy may be helpful in confirming the diagnosis of SD and identification of causative organism [33], it is an invasive technique with low sensitivity of identification of the causative organism (as low as 19%) [33]. CT-guided biopsy was not used in this study because all patients were already on empirical antibiotics prior to their admission at our hospital. This was expected to further decrease its ability to isolate the causative organism and further delay the treatment. Alternatively, a biopsy was acquired during surgery from all patients in this study.

The literature has thoroughly discussed the duration of postoperative antibiotic therapy, with multiple studies recommended that IV antibiotics to be administered for durations ranging from 10 days [24], 30 days [34] and up to 6 weeks [35], and oral agents duration ranges from 6 weeks [35] (after the end of IV course) up to 3 months [24], or even 6 months [36]. For economic and logistic reasons (difficulty of hospitalizing patients for long duration just to give IV antibiotics, and challenges of giving prolonged IV antibiotics at home), we limited the use of IV antibiotics to the period of hospital admission and oral antibiotic to 8 weeks, except for tuberculosis patients; our results were comparable to other studies that used IV/oral antibiotics for longer durations, which – along with good outcome of patients treated with empirical antibiotics when culture and sensitivity results were negative – points to the importance of efficient surgical technique in the outcome of management. A recently published study [37] confirmed that the postoperative use of oral antibiotics was as effective as intravenous antibiotics in the treatment of complex bone and joint infection. The choice of the antibiotic was tailored according to the results of histopathological examination and culture and sensitivity (when present). The empirical antibiotics used in this study was tailored to cover both *Staphylococcus aureus* (including methicillin-resistant strains MRSA) and Gram-negative bacteria, as the recent guidelines suggest [36, 38].

The presence of multiple co-morbidities has been recognized to be a major contributing factor in management failure and/or mortality [7]. In our study, 3 (7.7%) patients died during the follow-up period, all had many comorbidities. Other studies reported slightly lower mortality rates: 4.2% and 6.5% [7, 39], respectively. In these studies, patients had not been on empirical antibiotics preoperatively; which may point to the contribution of the faulty use of antibiotics to the outcome of this disease. We highly recommend careful preoperative assessment through the Spine Specialists, as well as controlling other associated medical issues through *multi-disciplinary team approach*, efficient surgical technique, tight postoperative follow-up that not only focuses on results of surgery, but also the one that addresses possible progress of accompanying chronic disease(s) and integrated management of multi-morbid patients with providers from other specialties. We believe that this approach can help improve the long-term outcome and survival rate of patients with this grave disease.

The short follow-up duration (minimum 6 months) is notable in this study. All patients were followed-up until complete resolution of their infection and 38 out of 39 patients did not show any sign of recurrence of their infection. However, this work focused on reporting the routine practice of our hospital and comparing it with the practice published in other studies from different spine centers rather than reporting the long-term follow-up of patients. Additionally, this series included both pyogenic and tuberculous SD, although they differ in causative organism and pathogenesis; the management of severe cases caused by either is similar. Other limitations in this study include heterogeneous group of patients (cervical, thoracic, lumbar) and varied surgical techniques used, together led to lack of variables amenable for comparison within the study.

## Conclusion

Surgical management of severe and complicated SD allows for effective debridement and rapid cure of inflammation, earlier patient mobilization and significantly shorter duration of antibiotic usage. Both tuberculous and pyogenic SD improved after sufficient surgery and postoperative *oral* antibiotics, which again points to the fact that the prolonged use of parenteral antibiotics is not mandatory in such cases as it has long thought to have been. Multidisciplinary approach in managing multimorbid patients with this disease, addressing other health issues, perioperatively as well as short and long-term follow-up can improve both management outcomes and long-term wellbeing and survival of these patients.

## Conflict of interest

The authors of this article declare that they have no conflict of interest affecting this study.
